# MdBZR1 and MdBZR1-2like Transcription Factors Improves Salt Tolerance by Regulating Gibberellin Biosynthesis in Apple

**DOI:** 10.3389/fpls.2019.01473

**Published:** 2019-11-27

**Authors:** Xuxu Wang, Xiude Chen, Qingjie Wang, Min Chen, Xiao Liu, Dongsheng Gao, Dongmei Li, Ling Li

**Affiliations:** ^1^College of Horticulture Science and Engineering, Shandong Agricultural University, Taian, China; ^2^State Key Laboratory of Crop Biology, Shandong Agricultural University, Taian, China; ^3^Yantai Institute of Coastal Zone Research, Chinese Academy of Sciences, Yantai, China

**Keywords:** brassinosteroids, gibberellins, MdBZR1, MdBZR1-2like, salt stress, *MdGA20ox2*, *MdGA3ox1*

## Abstract

Brassinosteroids (BRs) are a group of plant steroid hormones that play important roles in regulating plant development. In addition, BRs show considerable functional redundancy with other plant hormones such as gibberellins (GAs). BRASSINAZOLE RESISTANT1 (BZR1) and BRI1-EMS-SUPPRESSOR1 (BES1) transcription factors are negative feedback regulators of BR biosynthesis. This study provides evidence for the roles of *MdBZR1* and *MdBZR1-2like* in promoting GA production. These results also show that BRs regulate GA biosynthesis to improve salt tolerance in apple calli. Moreover, this research proposes a regulatory model, in which MdBZR1 and MdBZR1-2like bind to the promoters of GA biosynthetic genes to regulate their expression in a BR-dependent manner. The expression of key GA biosynthetic genes, *MdGA20ox1*, *MdGA20ox2*, and *MdGA3ox1* in yeast helps to maintain normal growth even under intense salt stress. In summary, this study underscores the roles of MdBZR1 and MdBZR1-2like in improving salt tolerance by regulating GA biosynthesis in apple calli.

## Introduction

Brassinosteroids (BRs) are sterol-derived phytohormones that regulate plant growth and development; their structures are similar to animal steroid hormones (Mitchell et al., 1970; Husna et al., 2018). The pathway of BR signaling from cell surface to the nucleus by transcription factors had been established by previous genetic and biochemical studies (Clouse, 2011; Wang et al., 2014). BR biosynthesis depends on a phosphorylation-dependent signaling pathway (Wang et al., 2012). In *Arabidopsis*, the BRASSINOSTEROID INSENSITIVE-1 (BRI1) receptor kinase functions at the first step of BR signaling. In the absence of BR, BRI1 is associated with BRI1 KINASE INHIBITOR-1 (BKI1) to inactivate BRI1. BR-INSENSITIVE-2 (BIN2) is a GSK3-like kinase that has been proposed to function in early BR signaling. In the absence of upstream BR signals, two BR-responsive transcription factors, BRASSINAZOLE RESISTANT-1 (BZR1) and BRI1-EMS-SUPPRESSOR-1 (BES1), are phosphorylated by BIN2, causing BZR1 and BES1 to be trapped in the cytoplasm due to the loss of their DNA binding abilities (Kim et al., 2018). In the presence of BRs, the binding of BR to BRI1 induces the phosphorylation of BRI1 by its receptor kinase BRI1-ASSOCIATED RECEPTOR KINASE1 (BAK1), leading to the disassociation of BKI1 and BRI1. This causes BRI1 to pass the signal to BR SIGNALING KINASE1 (BSK1) and CONSTITUTIVE DIFFERENTIAL GROWTH1 (CDG1) *via* direct phosphorylation, thereby promoting the binding of BRI1 SUPPRESSOR1 (BSU1) phosphatase to BIN2 (Kim et al., 2016). BSU1 then inactivates BIN2 through dephosphorylation, allowing BZR1/BES1 activation by PROTEIN PHOSPHATASE 2A dephosphorylation (Tang et al., 2011). BZR1/BES1 functions as a negative regulator of BR biosynthesis by feedback inhibiting *DWF*, *CPD*, *ROT3/CYO90D1*, and *BR6ox1/BR6ox2/OsBRD*, which are involved in BR biosynthesis (Wang et al., 2002; Yin et al., 2002; Zhao and Li, 2012).

Gibberellins (GAs) are mainly produced by the methylerythritol phosphate pathway from geranylgeranyl diphosphate (Kasahara et al., 2002). GA biosynthesis is regulated by GA20-oxidase (GA20ox), GA3-oxidase (GA3ox), and GA2-oxidase (GA2ox) (Lin et al., 2011). Trans-geranylgeranyl diphosphate is converted to GA_12_ aldehyde by an upstream GA biosynthetic pathway. GA20ox-overexpression in rice and citrus increases the level of bioactive GAs (Oikawa et al., 2004; Fagoaga et al., 2007). In addition, overexpression of the *Arabidopsis GA20ox* gene in kenaf leads to increased GA production, thereby enhancing the growth and fiber quality of kenaf (Withanage et al., 2015). Overexpression of *NtGA3ox* in tobacco influences GA content, suggesting that GA3ox plays an important role in maintaining GA homeostasis (Gallego-Giraldo et al., 2008).

Other genes—including many transcription factors—affect GA biosynthesis by regulating the expression of *GA20ox* and *GA3ox*. In tobacco, the bZIP transcription factor RSG influences GA biosynthesis by feedback regulating *NtGA20ox1* (Fukazawa et al., 2011). Overexpressing *AtIAA17* in *Arabidopsis* suppresses *AtGA3ox* transcription and leads to reduced level of bioactive GA_4_ (Shi et al., 2017). *AtWOX14* overexpression promotes *GA3ox* expression and suppresses *GA2ox* expression in *Arabidopsis*, leading to the accumulation of bioactive GAs (Denis et al., 2017).

BRs regulate cell development in many processes, including seed germination, stem elongation, seedling development, root growth, flower organ development, fruit development, and senescence (Kurepin et al., 2016). GAs exert profound and diverse functions on plant growth and development by regulating vegetative growth, flower induction, flower and fruit development, seed germination, and reserve mobilization (Hooley, 1994). BR deficiency has been shown to associate with the dwarf and deetiolated phenotypes in *Arabidopsis* (Clouse et al., 1996; Li et al., 1996), which are similar to those caused by the lack of bioactive GAs (Koornneef and Veen, 1980; Wilson and Somerville, 1995).

Interactions between hormones occur in various cell types and organs throughout the life cycle of plants. Likewise, the joint effect of different hormonal signals allows the plants to respond to various environmental changes (Grauwe et al., 2005). Similarly, BR signals coordinate with other hormonal signals to regulate endogenous developmental programs and help the plant to adapt to changing environments (Fridman and Savaldigoldstein, 2013). REPRESSOR OF ga1-3 (RGA) negative regulators of both the GA signaling pathways, BZR1 and RGA as mediators of signaling crosstalk between BRs and GAs, adjustment DELLAs in order to regulation of plant growth (Fukazawa et al., 2014). Previous studies in *Arabidopsis* have also shown that BRs control seed germination by regulating GA biosynthesis (Unterholzner et al., 2015).

Here, we report that MdBZR1 and MdBZR1-2like could bind to the promoters of both *MdGA20ox2* and *MdGA3ox1* and enhance their expressions in apple. Overexpression of MdBZR1 and MdBZR1-2like increased the GAs content in apple calli. Moreover, this study also found that the activities of GA20ox and GA3ox increased in response to salt stress. Salt stress negatively regulates GA biosynthesis and represses seed germination in soybean (Shu et al., 2017), whereas exogenous GA (GA_4+7_) application could promote the germination of *Leymus chinensis* seeds under salt stress (Wu et al., 2016). Here, this research probe into the molecular basis of how BR signaling regulates plant growth—the presence of BRs release MdBZR1 and MdBZR1-2like to upregulate the expression of *MdGA20ox2* and *MdGA3ox1*, which promotes GA biosynthesis and enhances salt tolerance in apple.

## Materials and Methods

### Plant Materials, Growth Conditions, and Treatment

Wild-type (WT) “Orin” apple calli were sub-cultured at 20 day intervals on Murashige and Skoog (MS) medium containing 0.5 mg·L^−1^ 6-benzylaminopurine and 0.5 mg·L^−1^ 2,4-dichlorophenoxy acetic acid at 25°C in the dark. The calli were subsequently used for genetic transformation and phenotypic analysis. The seeds of WT apple were used for the transient expression of *MdBZR1* and *MdBZR1-2like* genes.

Tissue-cultured WT plantlets of *Malus domestica* “Gala2” were sub-cultured monthly on MS medium supplemented with 0.5 mg·L^−1^ 6-benzylaminopurine and 0.2 mg·L^−1^ 3-indoleacetic acid at 25°C under a 16 h-light/8 h-dark photoperiod (the long-day condition). Twenty-day old sub-cultured apple seedlings were subjected to the 24-Epibrassinolide (EBR) and salt treatments. EBR is a highly active analog of the BRs (Wang et al., 2019) and is often used to test the response of plant tissues to BR exposure. We exposed tissue-cultured apple seedlings to four treatments for 15 days, including water (control), 100 nM EBR, 100 mM NaCl, and 150 mM NaCl.

### Identification of the Target Genes

The nucleotide sequences of the *MdBZR1* (GenBank accession number: MDP0000157809), *MdBZR1-2like* (GenBank accession number: MDP0000410792), *MdGA20ox1* (GenBank accession number: MDP0000280240), *MdGA20ox2* (GenBank accession number: MDP0000248981), and *MdGA3ox1* (GenBank accession number: MDP0000316943) genes were obtained by BLASTx searches against the *M. domestica* genome using the sequences of their homologous genes as query sequences in *Arabidopsis thaliana*.

### RNA Isolation and Quantitative Real-Time PCR (qRT-PCR)

Total RNA was extracted using a FastPure Plant Total RNA Isolation Kit (for polysaccharide- and polyphenol–rich tissues) (Vazyme Biotech Co., Ltd, Nanjing, China) with RNase-free Dnase (TaKaRa, Dalian, China) added to avoid DNA contamination. First-strand cDNAs were synthesized from 1 µg of RNA using a PrimeScript RT kit with gDNA Eraser (TaKaRa). Primer 3 (http://bioinfo.Ut.ee/primer3-0.4.0/) was used for primer design (Wang et al., 2018). Primer sequences of *MdBZR1*, *MdBZR1-2like*, *MdGA20ox1*, *MdGA20ox2*, *MdGA3ox1*, and *MdActin* (NCBI accession: EB136338), are shown in **Table S1**.

### Vector Construction and Plant Transformation

The full-length cDNAs of *MdBZR1*, *MdBZR1-2like*, *MdGA20ox1*, *MdGA20ox2*, and *MdGA3ox1* were used to construct over expression vectors. We constructed the *35S::BZR1-GFP* and *35S::BZR1-2like-GFP* recombinant vectors and transformed them into *Agrobacterium tumefaciens*. An empty *35S::GFP* vector was used as the control. Primers used for vector construction are shown in **Table S1**. We incubated 15-day-old apple calli with *Agrobacterium* thalli harboring the constructs for 30 min. Transgenic apple calli were identified by assessing the transient expression of GFP and samples testing positive for the presence of the overexpression vectors were used for further analysis.

The sequence of *MdBZR1* and *MdBZR1-2like* were amplified using primers MdBZR1-2like-IL60-F/R (**Table S1**). The resulting amplicons were cloned into the IL60-BS vector to generate the recombinant MdBZR1-IL60-2 and MdBZR1-2like-IL60-2 constructs. IL60-1 and MdBZR1-IL60-2 (MdBZR1-pIR) were co-transformed into apple seeds using a vacuum pump (FD-1D-50, BIOCOOL, Beijing, China), as were IL60-1 and MdBZR1-2like-IL60-2 (MdBZR1-2like-pIR. The qRT-PCR and gibberellic acid oxidase assays were performed on 5-day-old apple seedings after transiently expression *MdBZR1* and *MdBZR1-2like*.

### Electron Microscopy

Transgenic calli of WT in EBR treated, *35S::MdBZR1* and *35S::MdBZR1-2like* were cut into 1–2 mm^2^ pieces and examined under an electron microscope. All samples were fixed in 3.5% glutaraldehyde (prepared in a phosphoric acid buffer, pH 7.2) and washed with 0.1 M phosphate-buffered saline. The samples were then briefly post-fixed in 1% osmium tetroxide and dehydrated in an ascending ethanol series (10%–70% ethanol). Next, the samples were subjected to endosmosis and embedded and polymerized in Epon 812 resin. Ultra-thin sections were cut using an LKB-V ultramicrotome and stained with uranium acetate and lead citrate. Finally, ultrastructure of the samples were examined using a JEOL-1200EX tunneling electron microscope (TEM; JEOL, Tokyo, Japan) (Chen et al., 2018).

### HPLC-MS/MS Analysis of GAs Contents

In this experiment, endogenous GAs were extracted from the samples by isopropanol/water/hydrochloric acid, and the abundance of endogenous GA_1_, GA_3_, GA_4_, GA_5_, GA_6_, GA_7_, GA_8_, GA_9_, GA_13_, GA_14_, GA_15_, GA_19_, GA_20_, GA_24_, GA_29_, GA_44_, GA_51_, and GA_53_ was determined by 1290 UHPLC and mass spectrometry using the same equipment.

### Enzyme Activity Assays

The activities of superoxide dismutase, catalase, peroxidase (POD), ascorbate peroxidase (APX), glutathione reductase, GA20-oxidase (GA20ox), GA3-oxidase (GA3ox), and GA2-oxidase (GA2ox) in transgenic apple calli and seeds were quantified using a Plant Enzyme-linked Immunosorbent Assay Kit (Bangyi Biotechnology, Shanghai, China) following the manufacturer’s protocol.

### Malondialdehyde Assays

The method of Wang et al. was used for Malondialdehyde (MDA) measurements. Samples of 50 mg were homogenized in 1.8 ml 10% trichloroacetic acid and centrifuged for 20 min at 12,000×*g* (Wang et al., 2019). Then, 1 ml 10% trichloroacetic acid with 0.6% thiobarbituric acid was added to 1 ml of the supernatant. The mixture was heated in boiling water for 30 min, quickly cooled on ice, and centrifuged for 10 min at 1,600×*g*. The mixture’s absorbance at 532 (A532) and 600 nm (A600) was determined. Nonspecific absorbance at 600 nm was subtracted from A532. MDA concentration was calculated based on the adjusted A532 value with a MDA extinction coefficient of 155 mM^−1^ cm^−1^.

### Salt Tolerance Assays

For the salt tolerance assay, the tissue-cultured apple plantlets on MS medium supplemented with 0 mM NaCl, 100 mM NaCl, and 100 mM NaCl + 100 nM GA_3_ for 15 days grew. The triphenyl tetrazole chloride method was used to determine tissue viability (Towill and Mazur, 1975).

### *MdGA20ox1*, *MdGA20x2*, and *MdGA3ox1* Transformation

We transformed the pYES2-*MdGA20ox1*, pYES2-*MdGA20ox2*, and pYES2-*MdGA3ox1* constructs into *Saccharomyces cerevisiae* strain YPH500; the empty pYES2 vector was transformed as a control (An et al., 2018). The transformed strains of different concentrations were propagated on YPD medium supplemented with 0, 150, and 300 mM NaCl.

### Yeast One-Hybrid (Y1H) Assay

To conduct the Y1H assay, we first cloned the open reading frames of *MdBZR1* and *MdBZR1-2like* into the pGADT7 vector. The promoters of *MdGA20ox2 and MdGA3ox1* were cloned into the pHIS2 vector with their native promoters. We co-transformed four pairs of constructs, namely pGADT7-MdBZR1/pHIS-MdGA20ox2, pGADT7-MdBZR1/pHIS-MdGA3ox1, pGADT7-MdBZR1-2like/pHIS-MdGA20ox2, and pGADT7-MdBZR1-2like/pHIS-MdGA3ox1, into yeast strain Y187 according to the manufacturer’s instructions (Clontech, Beijing, China). The transformed yeast strains were grown on SD/-Leu/-Trp and SD/-Leu/-Trp/-His/260mM 3-amino-1,2,4-triazole (3-AT) plates.

### Promoter-GUS Analysis

The open reading frames of *MdBZR1* and *MdBZR1-2like* were cloned into the pGreenII 62-SK vector. Next, the promoter fragments of *MdGA20ox2 and MdGA3ox1* were cloned into the pCAMBIA1300-GUS vector. The two recombinant plasmids were then co-transformed into *Agrobacterium* thalli. Four combinations of constructs—35S-BZR1/35S-GA20ox2-GUS, 35S-BZR1/35S-MdGA3ox1-GUS, 35S-BZR1-2like/35S-GA20ox2-GUS, and 35S-BZR1-2like/35S-MdGA3ox1-GUS—were transformed into *Agrobacterium* thalli using a vacuum pump (FD-1D-50, BIOCOOL, Beijing, China). 35S/35S-GA20ox2-GUS and 35S/335S-GA3ox1-GUS were used as controls. The resulting apple calli were then cultured on solid MS media for 48 h at 25°C in the dark and histochemical staining was performed as previously described (Jefferson et al., 1987).

### Statistical Analyses

Each treatment was repeated three times on three independent biological replicates and the data are presented as mean ± SE. To detect statistically significant differences, we used ANOVAs with Duncan’s multiple range tests used for multiple comparisons. P-values < 0.05 were considered statistically significant. All statistical comparisons were performed using SPSS for Windows version 19 (IBM SPSS Inc., Chicago, IL, USA).

## Results

### BR Signaling Regulates the Transcript Levels of *MdGA20ox1*, *MdGA20ox2*, and *MdGA3ox1*

**Figure 1:**
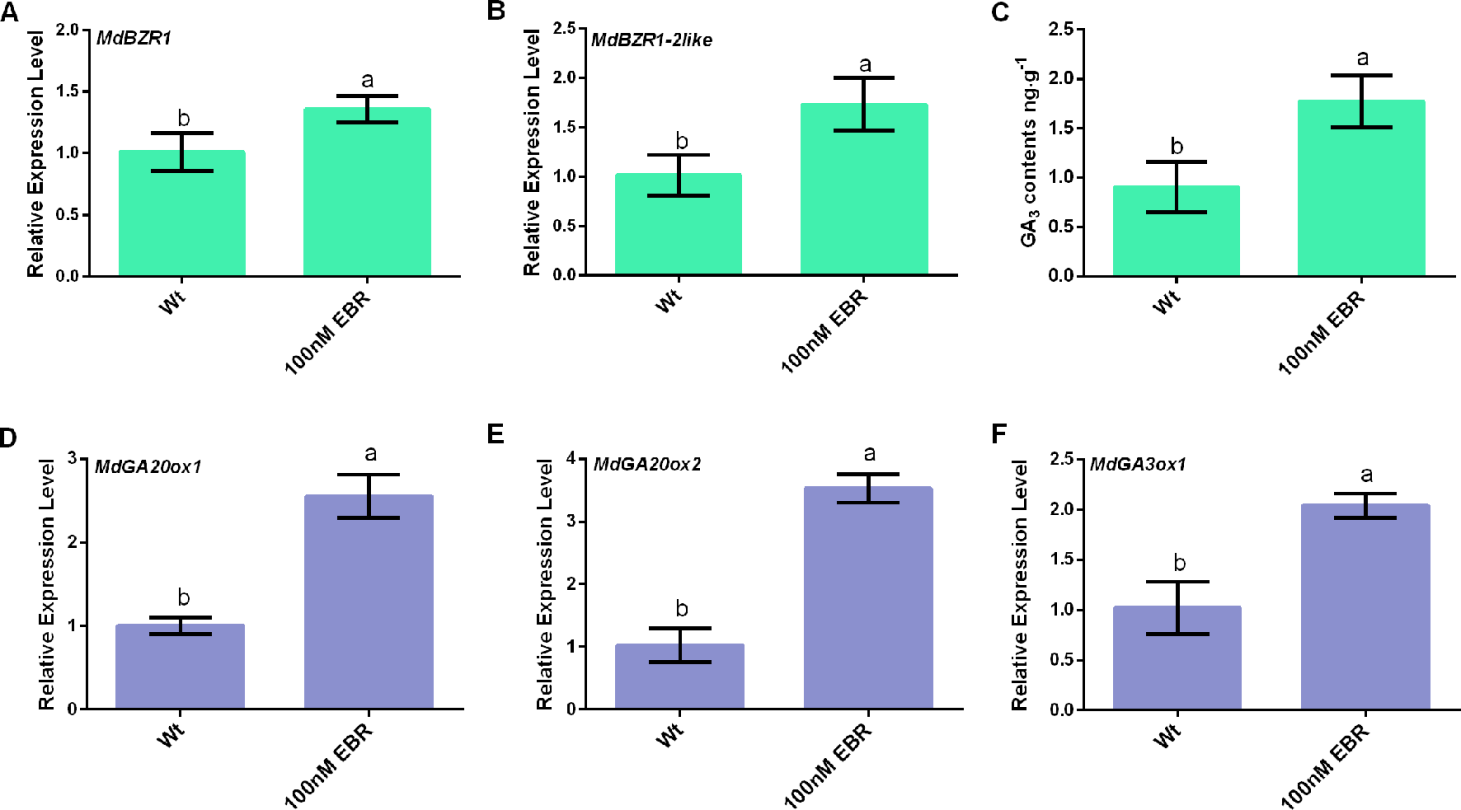
BR signaling associate with the upregulation of *MdGA20ox1*, *MdGA20ox2*, and *MdGA3ox1*. **(A)** *MdBZR1* relative expression level. **(B)** *MdBZR1-2like* relative expression level. **(C)** GA_3_ contents. **(D**–**F)** *MdGA20ox1*, *MdGA20ox2*, and *MdGA3ox1* relative expression levels. Data are shown as mean ± SE of three independent replicates. Different letters indicate significant differences at p 0.05.

In *Arabidopsis*, it has long been known that BRs and GAs function redundantly in many developmental programs, and the crosstalk between these two hormonal pathways occurs on the transcriptional level (Gallego-Bartolomé et al., 2012; Bernardo-García et al., 2014). Here, the result found that exogenously applied EBR led to increased *MdBZR1* and *MdBZR1-2like* transcript levels and GA_3_ content (**Figures 1A–C**). The transcript levels of *MdGA20ox1*, *MdGA20ox2*, and *MdGA3ox1* increased by over two folds in apple seedings treated by EBR compared with the non-treated control (**Figures 1D–F**). Taken together, these results suggest that that BR signaling presumably regulated *MdGA20ox1*, *MdGA20ox2*, and *MdGA3ox1* expression *via* the transcriptional control of *MdBZR1* and *MdBZR1-2like*.

### *MdBZR1* and *MdBZR1-2like* Overexpression Promotes Cell Elongation in Apple Calli

**Figure 2:**
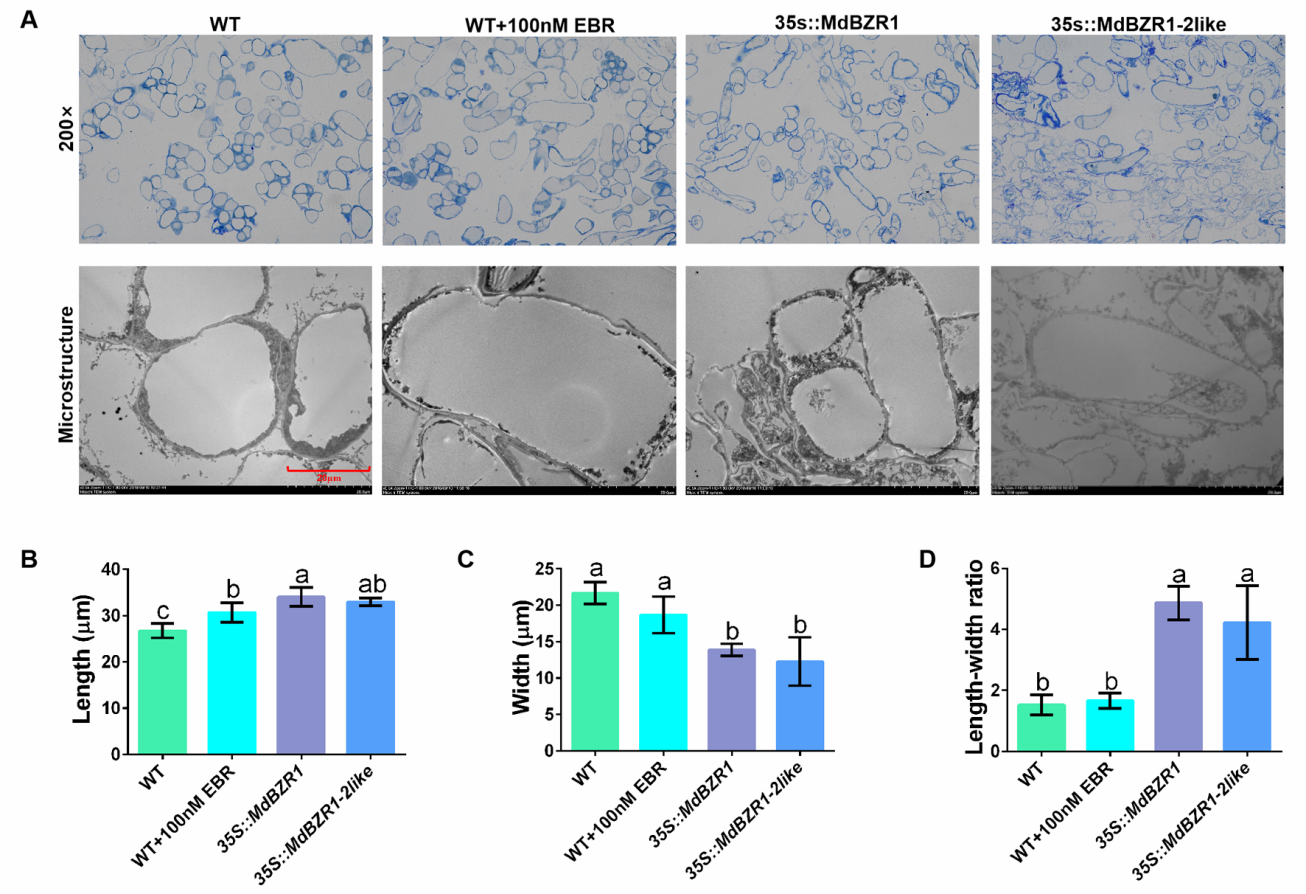
Cell elongation in *35S::MdBZR1* and *35S::MdBZR1-2like* calli **(A)** Microstructure of *35S::MdBZR1* and *35S::MdBZR1-2like* apple calli. **(B)** Longitudinal analysis of apple calli cells. **(C)** Transverse analysis of apple calli cells. **(D)** The length/width ratio of apple calli cells. Data are shown as means ± SE of three replicates. Different letters indicate significant differences at p < 0.05.

qRT-PCR and PCR were used to determine the transcript levels of *MdBZR1* and *MdBZR1-2like* in the three transgenic apple calli (**Figures S1A, B**). Morphology and cell microstructure analyses revealed promoted cell elongation in EBR-treated apple calli and transgenic apple calli compared with the WT (**Figure 2A**). Further measurements indicated that treatment with 100 nM EBR, *MdBZR1* overexpression, and *MdBZR1-2like* overexpression all had promoting effects on cell elongation (**Figures 2B, C**). The aspect ratio of *35S::MdBZR1* and *35S::MdBZR1-2like* apple calli was 2.5- and 2-fold greater than that of the WT, respectively (**Figure 2D**).

### MdBZR1 and MdBZR1-2like Promote GA Biosynthesis

The transcript levels of *MdGA20ox1*, *MdGA20ox2*, and *MdGA3ox1* increased in *35S::MdBZR1* transgenic apple calli (**Figure S2A**). In addition, the transcript levels of *MdGA20ox2* and *MdGA3ox1* increased in *35S::MdBZR1-2like* apple calli (**Figure S2B**). Previous work with *Arabidopsis* has demonstrated the involvement of GA20ox in the biosynthesis of GA_12_, GA_15_, GA_24_, and GA_9_ (Olszewski et al., 2002), whereas GA_1_ and GA_3_ are synthesized by GA3ox and then decomposed into GA_51_ by GA2ox (Olszewski et al., 2002). A total of 12 GAs in apple calli were identified (**Table 1**), and *MdBZR1* overexpression led to increased levels of GA_15_, GA_24_, GA_9_, GA_3_, GA_7_, GA_8_, GA_14_,and GA_19_, but a reduced level of GA_1_ compared with the WT, and the levels of GA_5_ and GA_6_ remained unchanged. Moreover, *35S::MdBZR1-2like* had increased levels of GA_24_, GA_9_, GA_51_, and GA_14_, and reduced the levels of GA_15_, GA_3_, GA_1_, and GA_5_; whereas GA_6_, GA_7_, GA_8,_ and GA_19_ levels were unchanged.

**Table 1:** GA levels in *35S::MdBZR1* and *35S::MdBZR1-2like* apple calli.

GA_s_	WT (ng·g^−1^)	*35S::MdBZR1* (ng·g^−1^)	*35S::MdBZR1-2like* (ng·g^−1^)
GA1	0.245 ± 0.02 a	0.045 ± 0.01 b	0.012 ± 0.01 c
GA3	0.015 ± 0.002 b	0.021 ± 0.001 a	0.0016 ± 0.001 c
GA5	0.660 ± 0.030 a	0.658 ± 0.249 a	0.251 ± 0.015 b
GA6	0.100 ± 0.041 a	0.123 ± 0.008 a	0.123 ± 0.016 a
GA7	0.001 ± 0.001 b	0.072 ± 0.005 a	0.002 ± 0.0007 b
GA8	0.129 ± 0.007 b	0.210 ± 0.032 a	0.115 ± 0.013 b
GA9	0.005 ± 0.001 b	0.080 ± 0.029 a	0.052 ± 0.006 a
GA14	0.027 ± 0.008 c	0.167 ± 0.005 a	0.059 ± 0.0005 b
GA15	0.015 ± 0.002 b	0.051 ± 0.003 a	0.002 ± 0.0004 c
GA19	0.124 ± 0.036 b	0.260 ± 0.023 a	0.091 ± 0.021 b
GA24	0.022 ± 0.007 c	0.041 ± 0.011 b	0.082 ± 0.0002 a
GA51	0.029 ± 0.008 b	0.058 ± 0.008 b	0.163 ± 0.063 a

Data are shown as means ± SE of three replicates. Different letters indicate significant differences at p < 0.05. GA, gibberellin; WT, wild-type.

**Figure 3:**
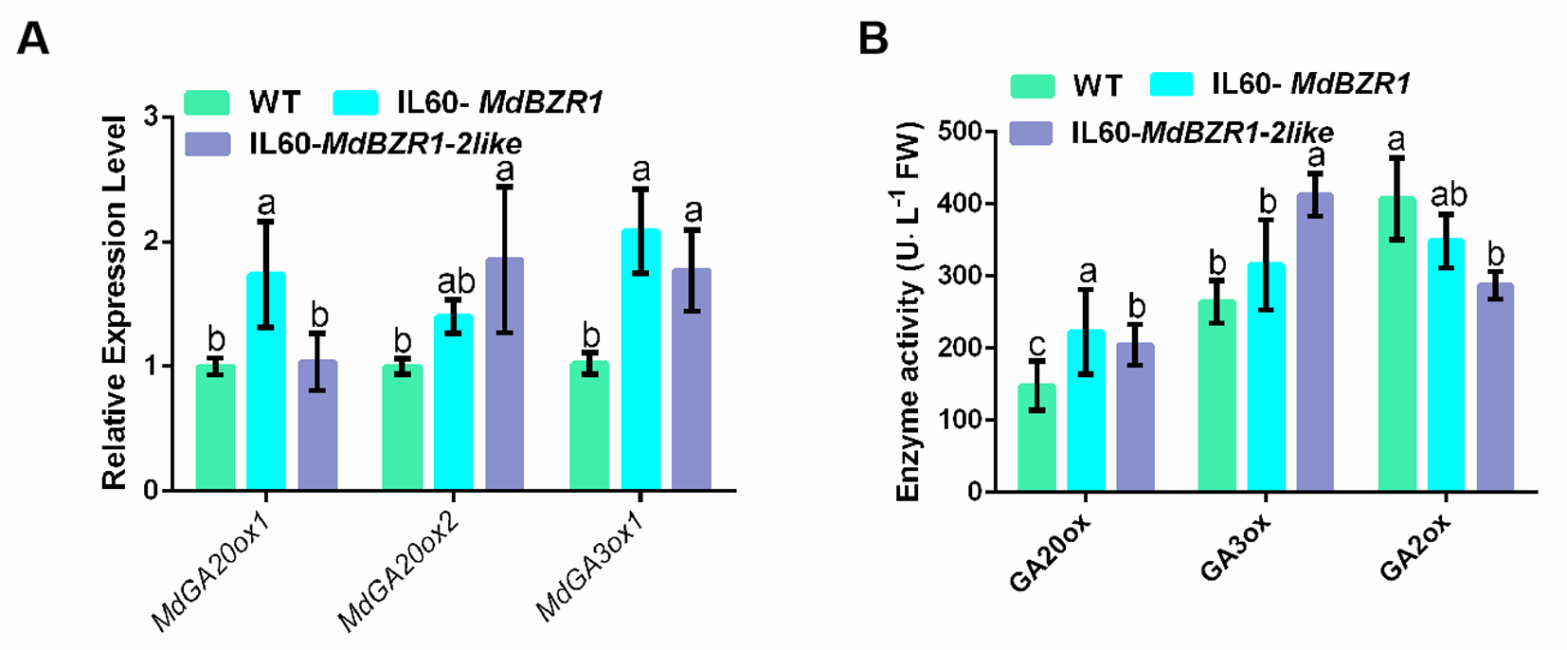
Transient overexpression of *MdBZR1* and *MdBZR1-2like* genes improves GA biosynthesis in apple seedings **(A)** The transcript levels of *MdGA20ox1*, *MdGA20ox2*, and *MdGA3ox1* in *35S::MdBZR1* and *MdBZR1-2like* apple seedings. **(B)** The activities of GA20ox, MdGA3ox, and MdGA2ox in apple seedings. Data are shown as means ± SE of three replicates. Different letters indicate significant differences at p < 0.05.

The results shown that the transcript levels of *MdGA20ox1* and *MdGA3ox1* in IL60-*MdBZR1* seedlings were upregulated; similarly, those of *MdGA20ox2* and *MdGA3ox1* were increased in IL60-*MdBZR1-2like* seedlings (**Figure 3A**). Consistently, GA20ox activity was improved in IL60-*MdBZR1* seedlings (**Figure 3B**). Specifically, the enzyme activities of GA20ox and GA3ox in IL60-*MdBZR1* and IL60-*MdBZR1-2like* seedings were both ∼1.5-fold higher compared with that of the control; whereas GA2ox enzyme activity was reduced in IL60-*MdBZR1-2like* seedlings (**Figure 3B**). A total of 14 GAs were detected in apple seedings (**Table 2**). The content of GA_3_, GA_5_, GA_6_, GA_8_, GA_9_, GA_13_, GA_14_, GA_19_, GA_24_, and GA_51_ in *35S::MdBZR1* seedlings were elevated than the WT. The GA_3_, GA_8_, GA_9_, GA_13_, GA_14_, GA_15_, GA_19_, GA_20_, GA_24_, and GA_51_ in *35S::MdBZR1-2like* seedlings were increased compared with the WT.

**Table 2:** GA contents in Il60-*MdBZR1* and IL60-*MdBZR1-2like* apple seedings.

GA_s_	WT (ng·g^−1^)	IL-60-MdBZR1 (ng·g^−1^)	IL-60-MdBZR1-2like (ng·g^−1^)
GA_1_	2.100 ± 0.191 a	2.000 ± 0.303 a	2.081 ± 0.045 a
GA_3_	0.016 ± 0.007 c	0.018 ± 0.001 b	0.030 ± 0.0002 a
GA_5_	0.040 ± 0.003 b	0.345 ± 0.026 a	0.045 ± 0.015 b
GA_6_	0.013 ± 0.0005 b	0.097 ± 0.002 a	0.002 ± 0.00005 b
GA_7_	0.487 ± 0.067 a	0.056 ± 0.001 b	0.042 ± 0.005 b
GA_8_	0.441 ± 0.127 c	1.364 ± 0.121 b	2.683 ± 0.088 a
GA_9_	0.032 ± 0.019 c	0.077 ± 0.029 b	0.371 ± 0.031 a
GA_13_	0.159 ± 0.004 c	0.464 ± 0.085 a	0.374 ± 0.012 b
GA_14_	0.048 ± 0.032 b	0.174 ± 0.028 a	0.192 ± 0.083 a
GA_15_	0.043 ± 0.012 b	0.002 ± 0.001 c	0.084 ± 0.006 a
GA_19_	0.149 ± 0.021 b	0.246 ± 0.017 a	0.119 ± 0.017 c
GA_20_	0.053 ± 0.003 b	0.022 ± 0.002 c	0.236 ± 0.027 a
GA_24_	0.078 ± 0.008 c	0.298 ± 0.085 a	0.267 ± 0.008 b
GA_51_	0.005 ± 0.001 c	0.040 ± 0.021 b	0.166 ± 0.045 a

Data are shown as means ± SE of three independent experiments. Different letters indicate significant differences at p < 0.05.

### MdBZR1 and MdBZR1-2like Bind to *MdGA20ox2* and *MdGA3ox1* Promoters

**Figure 4:**
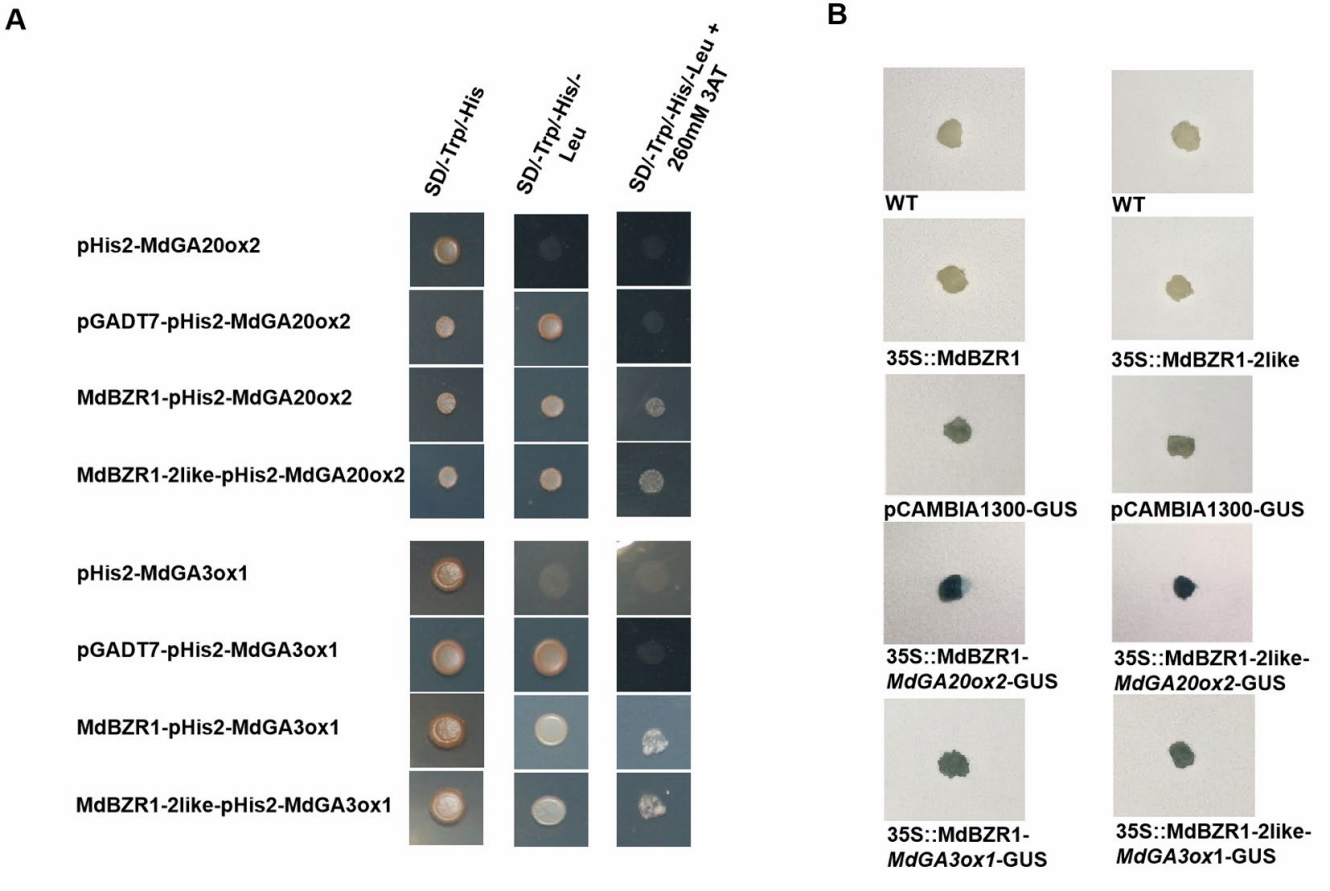
Interactions between MdBZR1, MdBZR1-2like and the promoters of *MdGA20ox2* and *MdGA3ox1 in vivo*. **(A)** Yeast One-Hybrid (Y1H) assay of the interaction between MdBZR1, MdBZR1-2like, and *MdGA20ox2* and *MdGA3ox1* promoters. The yeast strains were grown on SD/-Leu/-Trp and SD/-Leu/-Trp/-His/+260 mM 3AT (3-amino-1,2,4-triazole) medium. **(B)** GUS staining verification of the interactions between MdBZR1, MdBZR1-2like, and the promoters of *MdGA20ox2* and *MdGA3ox1* in apple calli.

To investigate how MdBZR1 and MdBZR1-2like regulate *MdGA20ox2* and *MdGA3ox1*, Y1H assay was performed. Yeast strains containing pGADT7-*MdBZR1*/pHIS2-*MdGA20ox2*, pGADT7-*MdBZR1*/pHIS2-*MdGA3ox1*, pGADT7-*MdBZR1-2like*/pHIS2-*MdGA20ox2*, or pGADT7-*MdBZR1-2like*/pHIS2-*MdGA3ox1* grew normally in the selective medium (**Figure 4A**), indicating that MdBZR1 and MdBZR1-2like could bind to the promoters of *MdGA20ox2* and *MdGA3ox1*. Results of the GUS-staining assay further validated this binding. The GUS staining of *MdBZR1*/*MdGA20ox2-*GUS, *MdBZR1*/*MdGA3ox1-*GUS, *MdBZR1-2like*/*MdGA20ox2-*GUS, and *MdBZR1-2like*/*MdGA3ox1-*GUS apple calli were darker than that of the control (**Figure 4B**).

### *MdGA20ox1*, *MdGA20ox2*, and *MdGA3ox1* Respond to Salt Stress

**Figure 5:**
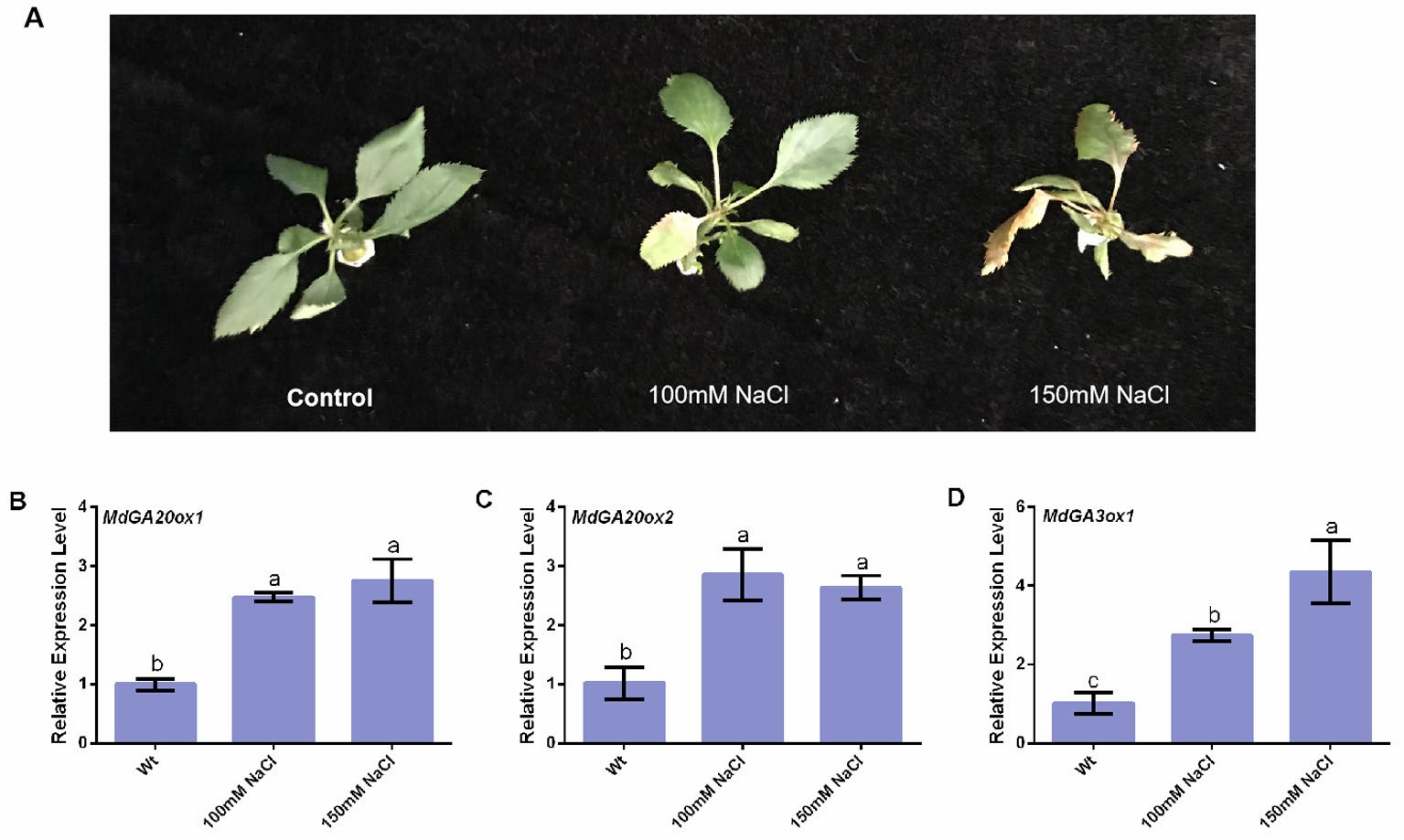
Responses of *MdGA20ox1*, *MdGA20ox2*, and *MdGA3ox1* to NaCl stress in wild-type “Gala2” tissue-cultured apple seeding. **(A)** Morphology of the treatment apple seedings under salt stress. **(B**–**D)** Transcript levels of *MdGA20ox1*, *MdGA20ox2*, and *MdGA3ox1* in “Gala2” tissue-cultured seeding under salt stress. Data are shown as means ± SE of three independent experiments. Different letters indicate significant differences at p < 0.05.

Tissue-cultured apple plantlets showed yellowing of leaves under salt stress (**Figure 5A**). Compared with the control, *MdGA20ox1*, *MdGA20ox2*, and *MdGA3ox1* had increased transcript levels in tissue-cultured apple seedlings in response to salt stress (**Figure 5B–D**).

### MdBZR1 and MdBZR1-2like Improve Salt Tolerance

**Figure 6:**
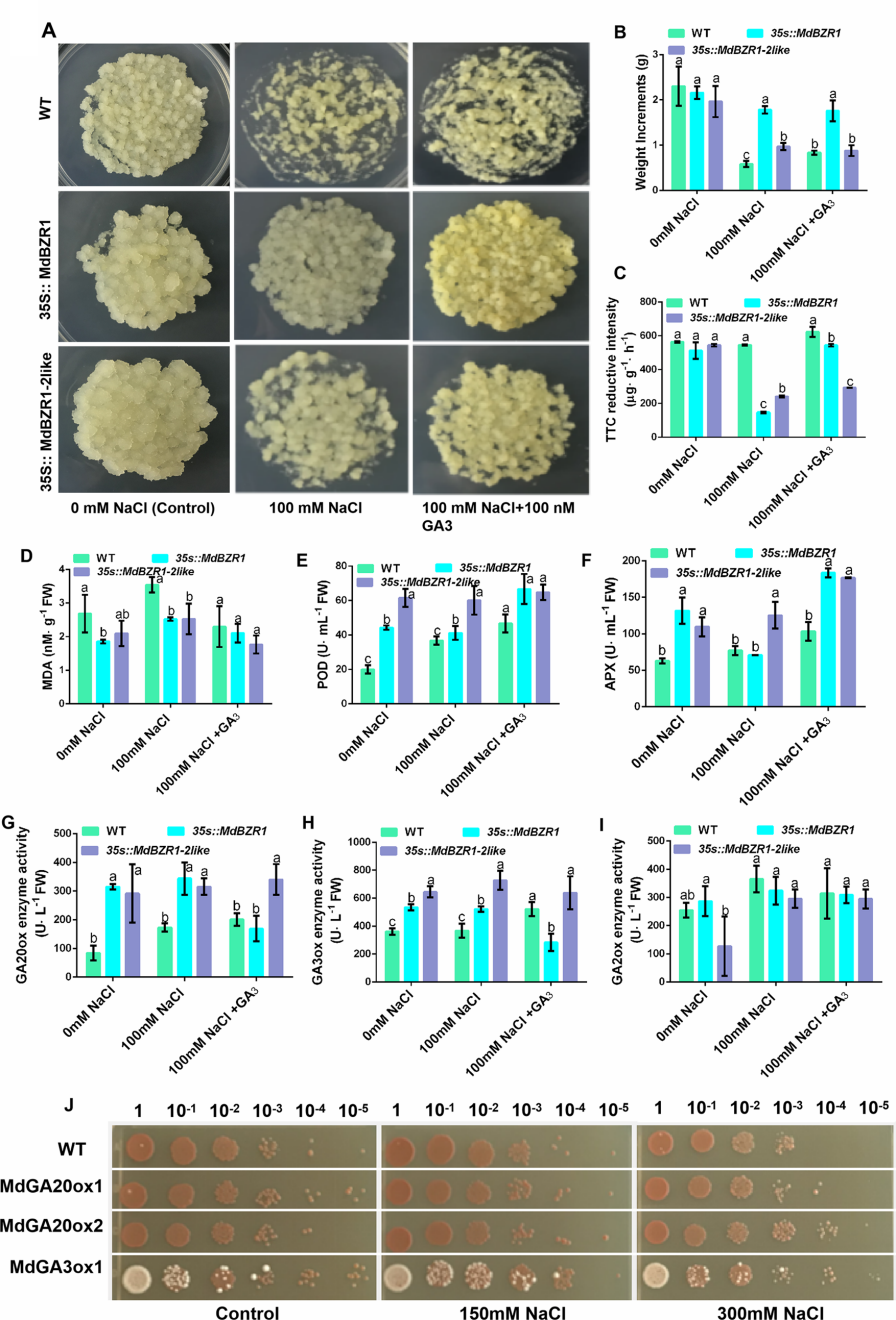
Phenotypic analysis of *35S::MdBZR1* and *35S::MdBZR1-2like* apple calli under salt stress. **(A)** Salt stress assays of *35S::MdBZR1* and *35S::MdBZR1-2like* apple calli. All calli were grown on medium containing 0 mM NaCl, 100 mM NaCl, and 100 mM NaCl + GA_3_ for 15 days. **(B)** Fresh weight of apple calli. **(C)** Triphenyl tetrazole chloride (TTC) reductive intensity in apple calli. **(D)** MDA concentration in apple calli. **(E)** Peroxidase (POD) activity in apple calli. **(F)** Ascorbate peroxidase (APX) activity in apple calli. **(G)** GA20-oxidase (GA20ox) activity in apple calli. **(H)** GA3-oxidase (GA3ox) activity in apple calli. **(I)** GA2-oxidase (GA2ox) activity in apple calli. **(J)** Effects of *MdGA20ox1*, *MdGA20ox2*, and *MdGA3ox1* overexpression on the growth of yeast strains in the presence of 0, 150, or 300 mM NaCl. Data are shown as means ± SE of three independent experiments. Different letters indicate significant differences at p < 0.05.

Both the WT and transgenic apple calli were grown on media containing of 0 mM NaCl, 100 mM NaCl, and 100 nM + GA_3_ for 15 days. The growth of transgenic apple calli was significantly more robust than that of the WT in NaCl stress (**Figure 6A**). The fresh weight of *35S::MdBZR1* and *35S::MdBZR1-2like* apple calli were significantly higher than that of the WT under 100 mM NaCl (**Figure 6B**). Interestingly, WT apple calli treated by NaCl + GA_3_ exhibited higher weight gain and triphenyl tetrazole chloride reductive intensity compared with those treated by NaCl alone (**Figures 6B, C**). MDA content is considered an indicator of stress level in plants (Wang at al., 2019). We found that the MDA concentration in WT apple calli treated by NaCl + GA3 and transgenosis apple calli in NaCl tretment were comparable to that without NaCl treatment (**Figure 6D**). POD and APX enzyme activities in *35S::MdBZR1*, *35S::MdBZR1-2like* apple calli were higher in NaCl treatment (**Figures 6E, F**). Similarly, the POD and APX of WT apple calli grown on NaCl + GA3 medium were also higher than those without NaCl (**Figures 6E, F**). By contrast, changes in superoxide dismutase, catalase and glutathione reductase activities were not significant (**Figure S3**).

As shown in **Figure 6G**, GA20ox activity in *35S::MdBZR1* and *35S::MdBZR1-2like* were higher in the presence than in the absence of NaCl. The enzyme activities of GA20ox and GA3oxs in WT apple calli were increased after NaCl + GA_3_ treatment (**Figures 6G, H**). However, GA3ox activity in *35S::MdBZR1* and *35S::MdBZR1-2like* calli were higher than that in the WT calli when treated by NaCl (**Figure 6H**). Moreover, GA2ox activity in the calli of the two transgenic lines and WT showed no significant difference under salt stress (**Figure 6I**). Finally, yeast strains carrying the pYES2-*MdGA20ox1*, pYES2-*MdGA20ox2*, and pYES2-*MdGA3ox1* constructs showed better growth than those transformed with the empty pYES2-vector (**Figure 6J**). Taken together, these results suggested that *MdBZR1* and *MdBZR1-2like* improving salt resistance presumably by regulated GA biosynthesis in a way.

## Discussion

### GA Biosynthesis Is Regulated by BZR1 and BZR1-2like

The BR-deficient *bri1* mutant exhibits dwarfism when grown under the light and etiolation when grown in the dark. This is presumably because the upstream BR signal and/or the *bzr1-1D* mutation promote cell elongation in the dark. When growing in the light, these plants show a dwarf phenotype that features short hypocotyls and petioles (Clouse et al., 1996; He et al., 2005). In general, the phenotypes of BR-deficient mutant resemble plants lacking sufficient GAs (Wilson and Somerville, 1995). A recent study has found that the transcript level of *GA20ox1* reduced in *bri1-1* but increased in *bzr1-1D* (Gallego-Bartolomé et al., 2012). Other studies have shown that in rice and *Arabidopsis*, BRs could induce *GA20ox* expression and regulate GA biosynthesis (Stewart Lilley et al., 2013; Tong et al., 2014). In line with these published data, we found that the transcript levels of *MdBZR1*, *MdBZR1-2like*, *MdGA20ox1*, *MdGA20ox2*, and *MdGA3ox1*, as well as the content of GA_3_ were elevated upon EBR application (**Figure 1**). Similarly, *MdGAoxs* expression also increased by various degrees in *35S::MdBZR1* and *35S::MdBZR1-2like* calli compared with the WT (**Figure S2**). These results suggest a crosstalk between BR signaling and GA biosynthesis in apple, mediated by the binding of MdBZR1 and MdBZR1-2like to *MdGA20ox2* and *MdGA3ox1* promoters, thereby promoting GA biosynthesis (**Figures 2**–**4**).

Previous studies have shown that BES1 and BZR1 bind to E-box motifs (5′-ACnnGT-3′), G-box motifs (5′-CACGTG-3′), BRRE elements (5′-GCTG(T/C)G-3′), and non-E-box motifs (5′-AA(A/T)CAAnnnC(C/T)T-3′) in *Arabidopsis* (Sun et al., 2010; Yu et al., 2011; Unterholzner et al., 2015). However, the binding of MdBZR1 and MdBZR1-2like to the functional elements in *MdGA20ox2* and *MdGA3ox1* promoters warrants further study.

### GA-BR Crosstalk Improves Salt Tolerance

Studies have shown that the BZR1 transcription factors regulate FER2- and FER3-mediated reactive oxygen species signaling, thereby increasing heat stress tolerance in tomatoes (Yin et al., 2018). In *Brassica rapa*, BZR-related genes are implicated in drought, cold, and salt stress responses (Saha et al., 2015). Furthermore, in *Brassica napus*, the crosstalk between BR and jasmonic acid pathways affects the regulation of plant growth, as jasmonic acid-related genes regulate cell wall composition and stress responses through BR signaling genes, such as BZR1/BES1 (Sahni et al., 2016). Consistently, we found that the overexpression of *MdBZR1* and *MdBZR1-2like* promote salt resistance in ‘Orin’ apple calli (**Figure 6**).

The NaCl treatment significantly increases the expression of *SlGA20ox1*, *SlGA3ox1*, and *SlGA3ox2* in tomato seeds (Nakaune et al., 2012). Similarly, we found that exogenously applied NaCl could increase the transcript levels of *MdGA20ox1*, *MdGA20ox2*, and *MdGA3ox1* in tissue-cultured apple seedlings (**Figure 5**). Under salt stress, we observed enhanced GA20ox and GA3ox activities in *35S::MdBZR1* and *35S::MdBZR1-2like* apple calli (**Figure 6**). GA20ox and GA3ox promote GA biosynthesis (Hedden and Thomas, 2012). However, exogenous GA application has been reported to increase the germination rate of *Reaumuria soongorica* seeds under salt stress (Niu et al., 2017). Similarly, we observed improved salt tolerance in *35S::MdBZR1*, *35S::MdBZR1-2like*, and WT apple calli upon GA_3_ application (**Figure 6**). The *MdBZR1* and *MdBZR1-2like* genes expression levels and content of GA_3_ in apple calli with EBR were increased (**Figure 1C**). The levels of GAs, including GA_3_, were also increased in *35S::MdBZR1* or *35S::MdBZR1-2like* transgenic apple calli (**Tables 1** and **2**). Moreover, the growth of *MdGA20ox1*-, *MdGA20ox2*-, and *MdGA3ox1*-transformed yeast strains were notably more robust compared with the control in NaCl-containing medium (**Figure 6G**). Taken together, these results suggest that *MdBZR1* and *MdBZR1-2like* overexpression *could* moderately improve salt tolerance may in a *MdGA20ox2*- and *MdGA3ox1*-dependent manner.

Moosavi had reported that GA induces the defense response in plants and POD activity increased 4 weeks following the application (Moosavi, 2017). Similarly, our results show that GA could stimulate the innate anti-oxidation defense responses and improve the resistance of tomato plant against *Meloidogyne javanica* damage. The interaction between ascorbic acid and GA_3_ prevents the decrease in lipid peroxidation and enhances the activities of antioxidant enzymes, improving the Ni tolerance of soybean seedlings (Saeidi-Sar et al., 2007). Our findings are in line with previous studies—the GA + NaCl treatment led to decreased MDA content but improved POD and APX activities in *35S::MdBZR1*, *35S::MdBZR1-2like*, and WT apple calli (**Figure 6**). Future study should focus on how GA stimulates the innate anti-oxidation defense responses against salt stress.

## Conclusions

**Figure 7:**
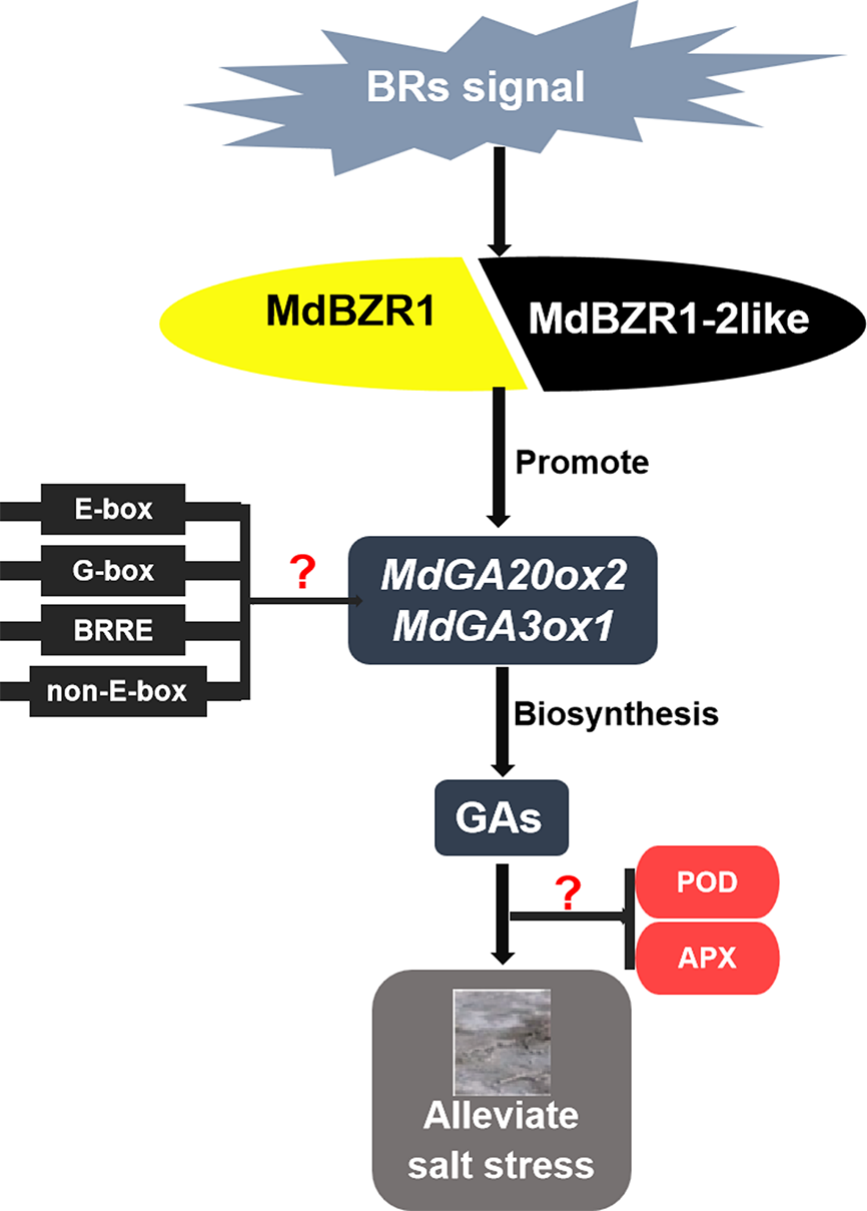
Proposed model for the mechanism of MdBZR1 and MdBZR1-2like in regulating gibberellin biosynthesis to improve salt tolerance. Question marks indicate sections lacking experimental evidence.

In summary, we found that both MdBZR1 and MdBZR1-2like could bind to the promoters of *MdGA20ox2* and *MdGA3ox1*, thereby regulating GA biosynthesis to alleviate the damage caused by salt stress in apple (**Figure 7**).

## Data Availability Statement

This manuscript contains previously unpublished data. All datasets generated for this study are included in the article/**Supplementary Material**.

## Author Contributions

XW performed the experiments and wrote the manuscript. XC, QW, MC, and XL provided technical and theoretical supports. DG, DL, and LL supervised the project and provided the fundings.

## Funding

We thank the National Natural Science Foundation of China (grant no. 31872041 and 31601706) and the Natural Science Foundation of Shandong Province (grant no. ZR2018MC023) for supporting this work.

## Conflict of Interest

The authors declare that the research was conducted in the absence of any commercial or financial relationships that could be construed as a potential conflict of interest.
