# Supplementary material for: MdBZR1 and MdBZR1-2like Transcription Factors Improves Salt Tolerance by Regulating Gibberellin Biosynthesis in Apple
Source: Front Plant Sci. 2019 Nov 27;10:1473. doi: 10.3389/fpls.2019.01473 (PMC6892407; doi:10.3389/fpls.2019.01473)
Supplement: Supplementary file 1 [file DataSheet_1.pdf]

SUPPLEMENTARY MATERIAL

Table S1. Primers used in this study.

Gene	Expression vector	Forward	Reverse
MdBZR1	pRI101-GFP	CATATGATGACGTCGG ATGGGGCGAC TT	GGATCCAATCCGAGCC TTTCCATTCC CAAGC
	pGADT7	CATATGATGACGTCGG ATGGGGCGAC TT	GGATCCAATCCGAGCC TTTCCATTCC CAAGC
	pGreenII 62-SK	CATATGATGACGTCGG ATGGGGCGAC TT	GGATCCAATCCGAGCC TTTCCATTCC CAAGC
	qRT-PCR	CTATCTCCGCTCTGCAATCC	GAGACGGCGTAAAATGGGTA
MdBZR1-2like	pRI101-GFP	CATATGATGACAGGCG GTGGTTCATC	GGATCCATGGTTCTTC CCATTGCCAA GTGTG
	pGADT7	CATATGATGACAGGCG GTGGTTCATC	GGATCCATGGTTCTTC CCATTGCCAA GTGTG
	pGreenII 62-SK	CATATGATGACGTCGG ATGGGGCGAC TT	GGATCCAATCCGAGCC TTTCCATTCC CAAGC
	qRT-PCR	GCTACAAGCTTCCCAAGCAC	TTGGGAAAGAGGAGGAGGAT
MdGA20ox1	pYES2	GGATCC ATGGCTGTTG AGTGCATGAT	TCTAGACAGTTTTTGG TTGCTTTTCT G
	qRT-PCR	CTCTCCGGTGACAAAGAAGC	GGAGAATCTGCCAGTGAAGC
MdGA20ox2	pYES2	GGATCC ATGGCTGTTG AGTGCATGAT C	TCTAGA CAGTTTTTGG TTGGTTTTCT GTTG
	pHIS2	GAATTCTCAATAGAAT ATGTCTCTTC TCTTG	GAGCTCTTTTGCATGA ACAGAAAATG
	pCAMBIA1300-GUS	GTCGACTCAATAGAAT ATGTCTCTTC TCTTG	GGATCCTTTTGCATGA ACAGAAAATG
	qRT-PCR	AAGCCTCGCAACTTGTAGGA	GGAGAATCTGCCAGTGAAGC
MdGA3ox1	pYES2	GAGCTCATGTCGAGTG GAAAACTCTC AG	TCTAGAATCTAGAGGA GCATTCAGTC GGA
	pHIS2	GAATTCATCTCATRAG CCTTCTTYGC GAA	GAGCTCAGTAGATAAT AGTAATACGA GAGAGGG
	pCAMBIA1300-GUS	GTCGACATCTCATRAG CCTTCTTYGC GAA	GGATCCAGTAGATAAT AGTAATACGA GAGAGGG
	qRT-PCR	CCATCGCATATCTGCCTTTT	GCTGCAAGCCTTTTCATCTC
MdActin	qRT-PCR	TACTCAGCTTTGGCAATCCACATC	TGACCGAATGAGCAAGGAAATTACT

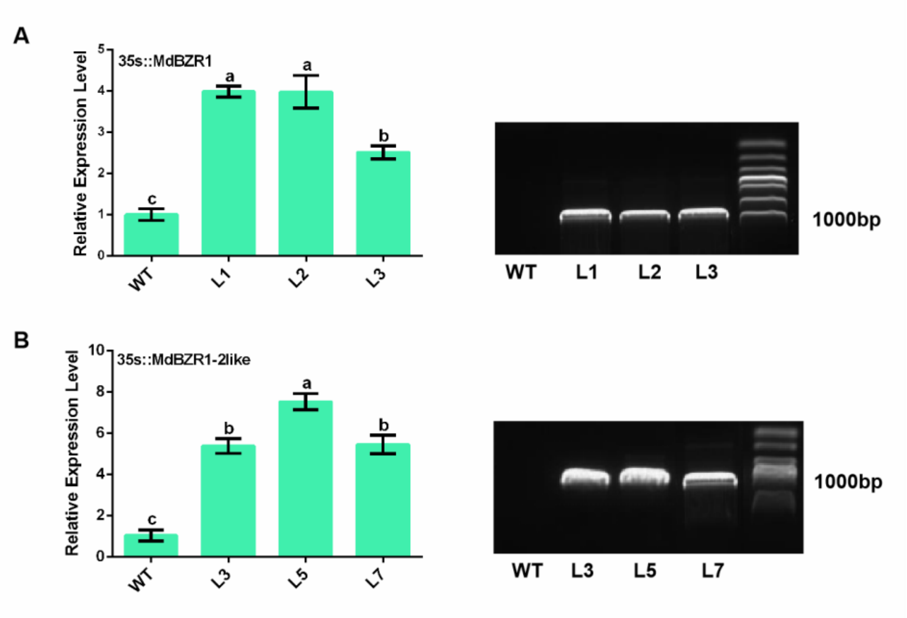

Fig S1. qRT-PCR and PCR analyses of *MdBZR1* and *MdBZR1-2like* in transgenic apple calli. (A) Identification of the *35S::MdBZR1* apple calli. (B) identification of the *35S::MdBZR1-2like* apple calli. Data are represented as means ± SE of three independent experiments. Different letters indicate significant differences at $p < 0.05$.

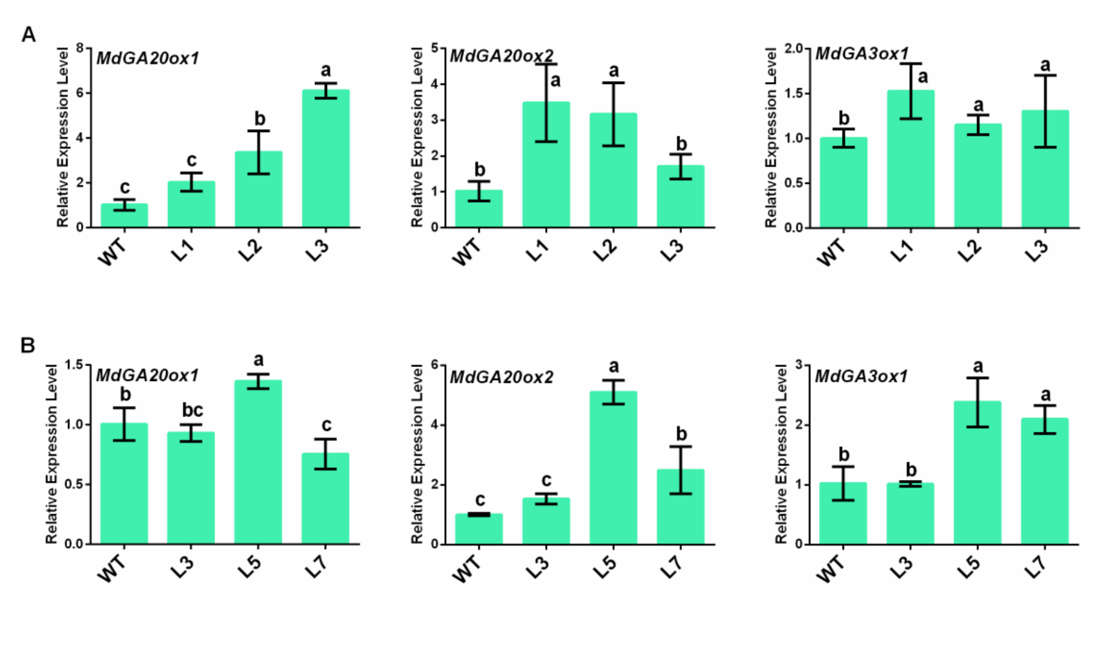

Fig S2. The transcript levels of *MdGA20ox1*, *MdGA20ox2*, and *MdGA3ox1* in *35S::MdBZR1* and *35S::MdBZR1-2like* apple calli. (A) The transcript levels of *MdGA20ox1*, *MdGA20ox2*, and *MdGA3ox1* in *35S::MdBZR1* apple calli. (B) The transcription levels of *MdGA20ox1*, *MdGA20ox2*, and *MdGA3ox1* in *35S::MdBZR1-2like* apple calli. Data are shown as means ± SE of three independent experiments. Different letters indicate significant differences at $p < 0.05$.

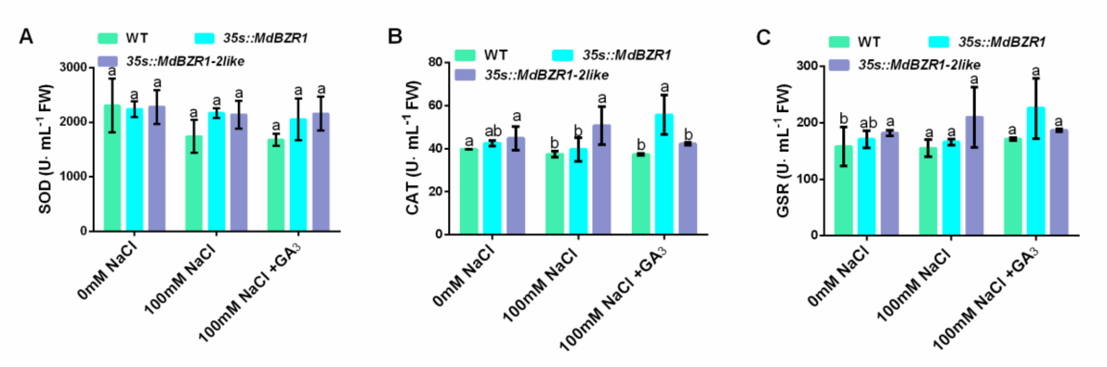

Fig S3. The activity of antioxidant enzymes in *35S::MdBZR1* and *35S::MdBZR1-2like* apple calli under salt stress. (A) SOD activity. (B) CAT activity. (C) GSR activity. Data are shown as means ± SE of three independent experiments. Different letters indicate significant differences at $p < 0.05$.
